# Tube erosions following glaucoma drainage device implantations

**DOI:** 10.1038/s41598-025-08282-x

**Published:** 2025-07-18

**Authors:** Julia Prinz, Karl Mercieca, Laura-Jill Förster, Kira Hilmers, Peter Walter, Matthias Fuest, Björn Bachmann, Claus Cursiefen, Verena Prokosch

**Affiliations:** 1 Department of Ophthalmology, RWTH Aachen University, 52074 Aachen, Germany; 2 Department of Ophthalmology, Faculty of Medicine, University Hospital of Cologne, 50937 Cologne, Germany; 3 Department of Ophthalmology, University of Bonn, 53117 Bonn, Germany

**Keywords:** Glaucoma, Refractory glaucoma, Intraocular pressure, Glaucoma drainage devices, Fascia lata, Medical research, Clinical trial design, Ocular hypertension, Glaucoma

## Abstract

Glaucoma drainage devices (GDD) are an important treatment option for advanced and complex glaucoma patients. To prevent tube erosion, different materials may be used to patch the tube. The aim of this study was to compare tube erosion rates of allogenous fascia lata versus corneal stromal patches relating to Ahmed glaucoma implant (AGI) and Paul glaucoma implant (PGI) surgeries. In this retrospective study, 84 patients were included. The tube was covered with allogenous fascia lata (*n* = 43) or a corneal stromal patch (*n* = 41). 32 eyes of 31 patients underwent AGI and 52 eyes of 52 patients underwent PGI surgeries. The number of tube erosions was evaluated during 18 months of follow-up. Tube erosions occurred in 4 patients with fascia lata patches (9.3%) and 1 patient (2.4%) with a corneal stromal patch (*p* = 0.184). In the superior quadrants (*n* = 78; 92.9% of all GDD), tube erosions were significantly more frequent with fascia lata (*n* = 4) compared to corneal stromal (*n* = 0) patches (*p* = 0.045). There was no difference in the number of tube erosions between the AGI (*n* = 2) and PGI (*n* = 3) group (*p* = 0.928). Tube erosions after GDD are rare. Fascia lata patches were more frequently associated with tube erosions than corneal stromal patches.

## Introduction

Glaucoma is a leading cause of irreversible blindness^1^. The global prevalence of glaucoma in the population aged between 40 and 80 years is 3.5%^2,3^. Lowering intraocular pressure (IOP) is the only proven treatment strategy^4^. Glaucoma drainage devices (GDD) are a well-established option in the management of refractory glaucoma^5^. GDD may be considered as primary glaucoma surgery in patients with conjunctival scarring due to prior ocular surgery, cicatricial conjunctival diseases or anterior segment dysgenesis. In certain clinical contexts such as neovascular, uveitic, congenital, or aphakic glaucoma, GDDs may also be considered when trabeculectomy is less likely to succeed or contraindicated due to ocular surface pathology or uncontrolled inflammation^6,7^. GDD consist of a tube which facilitates aqueous humour flow from the anterior chamber into a post-equatorial, subconjunctival base plate^8^. According to the multicenter randomized *Primary Tube versus Trabeculectomy* (PTVT) and *Tube versus Trabeculectomy* (TVT) studies, GDD were associated with a similar IOP reduction compared to trabeculectomy after 5 years of follow-up^9,10^. The TVT study showed a higher success rate in the GDD compared to the trabeculectomy group as well as a higher incidence of early postoperative complications after trabeculectomy than GDD^11^.

However, tube erosion through the conjunctiva is a significant complication following GDD implantation and may lead to hypotony and endophthalmitis^12^. The incidence of tube erosions after GDD implantations ranges from 0 to 12% in previous studies^12–17^. Measures to reduce the risk of conjunctival erosion and tube exposure include covering of the tube intraoperatively by an autologous partial thickness scleral flap^18^. However, external or internal erosion of the tube may still occur, especially in eyes having previously undergone multiple surgeries^19,20^. In 1987, Freedman introduced tube coverage with allogenous sclera^21^. Since then, various patch materials, such as allogenous fascia lata, cornea, dura mater, or bovine pericardium have been used to cover the part of the tube that lies on the sclera^15,22–24^. To date, there is no consensus on which patch material might be superior in terms of durability, safety, or biocompatibility.

The Paul glaucoma implant (PGI; Advanced Ophthalmic Innovations, Singapore, Republic of Singapore) is a novel GDD with smaller external tube diameter (467 μm) compared to other GDD, such as the Ahmed glaucoma implant (AGI, New World Medical, Rancho Cucamonga, California, USA, external tube diameter 635 μm), which hypothetically reduces the risk of tube erosion^25^.

In this study we investigated the incidence of tube erosions using allogenous fascia lata or corneal stromal patches covering the tubes of the AGI and PGI during 18 months of follow-up.

## Materials and methods

### Patient characteristics

In this retrospective study, 84 patients were included. Between October 2020 and December 2022, 32 eyes of 31 patients underwent AGI and 52 eyes of 52 patients underwent PGI. The tube was covered with an allogenous single-layered fascia lata (*n* = 43) or corneal stromal patch (*n* = 41). The 12- and 18-month follow-up appointments were attended by 21 and 18 patients and 40 and 22 patients in the AGI and PGI groups respectively. Intraoperatively, the part of the tube that lies on the sclera was covered with allogenous Tutoplast fascia lata (fascia lata, Bess Medizintechnik GmbH, Berlin, Germany) or anterior lamellae from allogenous corneas which were obtained from the corneal bank of the Department of Ophthalmology, University of Cologne, Germany (cornea). Patients underwent clinical examination preoperatively and at 1 day, 1 week, 6 weeks, 3, 6, 12, and 18 months postoperatively. Preoperatively, data on age, sex, and ocular history were recorded. All patients underwent surgery at the Department of Ophthalmology at the University Hospital of Cologne. All surgeries were performed by the same experienced surgeon (V.P.). The study adhered to the tenets of Helsinki. It was approved by the medical ethics committee of the University of Cologne (21-1539). Informed consent was obtained from all participants prior to their inclusion in the study.

### Surgical technique

The procedure of GDD implantation has been described previously^26^. The surgeries were performed in general or sub-Tenon’s anaesthesia. The most common surgical sites were the supero-temporal and supero-nasal quadrants. In cases of excessive scarring of the superior quadrants, the GDD implantation was performed in the infero-nasal quadrant. A corneal traction suture (7 − 0 silk, Ethicon, Johnson & Johnson Medical GmbH, Norderstedt, Germany) was placed to pull the eye into down- or upgaze. Following a conjunctival and Tenon’s peritomy, light cautery was applied to the scleral bed. Three non-fragmenting polyvinyl alcohol shields of 4 × 4 mm (ProOphtha Sponges, Lohmann & Rauscher, Neuwied, Germany) soaked with mitomycin C 0.5 mg/ml were applied subconjunctivally extending approximately 2 mm posterior to the limbus distributed evenly, including the peripheral scleral region involved in the surgical field for 3 min. Then, this area was irrigated with balanced salt solution (Alcon BSS, Alcon Pharma GmbH, Freiburg, Germany). The plate of the GDD was then placed 8–10 mm from the limbus (for PGI: beneath the recti muscles) and sutured to the sclera using 8 − 0 non-absorbable nylon (Ethicon) sutures. For PGI, a part of a 6 − 0 non-absorbable monofilament polypropylene suture (Prolene, Ethicon) was inserted into the tube to reduce immediate post-operative flow and prevent postoperative hypotony. The tube was cut to an estimated length to allow for a short length within the anterior chamber. The intraluminal suture was pushed to the tip of the tube. Then, a deep scleral horizontal tunnel was created using a bent 23 (AGI) or 26 Gauge (PGI) needle and the tip of the tube was guided through the tunnel into the anterior chamber. When flow through the tube was confirmed at the plate end, the tube was sutured to the sclera using an 8 − 0 nylon ‘box’ suture (Ethicon). For PGI, the distal end of the intraluminal suture was placed into a subconjunctival pocket.

A patch of allogenous fascia lata or cornea was cut to fit over the length of the part of the tube that lies on the sclera (approximately 6 × 6 mm) and was then sutured to the scleral bed using 10 − 0 nylon sutures (Ethicon). The corneal patch grafts were prepared by trephining a central button from donor corneal tissue, deliberately avoiding the limbal area to exclude stem cell–rich zones and vascularized tissue. The corneal stromal patches were obtained after Descemet stripping for Descemet Membrane Endothelial Keratoplasty (DMEK) and therefore consisted of corneal stroma and epithelium only^27^. During implantation, the epithelial side was oriented upward, while the stromal surface was placed directly over the drainage tube to ensure optimal conformity and coverage.

The fascia lata grafts were oriented so that its parallel fibres were placed perpendicular to the tube’s longitudinal axis in line with the technique described by Tanji et al., aiming to minimize the risk of graft splitting and subsequent tube exposure^22^. Finally, the conjunctiva was closed with Vicryl 8 − 0 (Ethicon).

Following both AGI and PGI, patients were treated with dexamethasone 1 mg/ml eye drops (Dexa-sine SE, Alcon Pharma GmbH, Freiburg, Germany) 8 times daily for 1 week tapering it down by one drop a week thereafter. Ofloxacin 3.0 mg/ml (Floxal EDO, Dr. Gerhard Mann chem.-pharm. Fabrik GmbH, Berlin, Germany) eye drops were applied 4 times daily for 1 week.

### Statistical analysis

Statistical analysis was performed using the Statistical Package for Social Sciences (IBM SPSS Statistics for Windows, Version 25, Armonk, NY: IBM Corp.). All values are displayed as mean ± standard deviations. Differences between groups were compared using independent-group t-tests. Proportions for categorical variables were compared using the chi-squared test. A p-value of < 0.05 was considered statistically significant.

## Results

There were no significant differences in the distribution of age (*p* = 0.589), gender (*p* = 0.383), or glaucoma subtypes between the corneal stromal and fascia lata patch group (Table 1). The numbers of previous ocular surgeries of all patients are displayed in Table 2. Altogether, 78 GDD (92.9%) were positioned in the supero-temporal or supero-nasal quadrant. A total of 40 eyes (93.0%) in the fascia lata group and 38 eyes (92.7%) in the corneal stroma group were implanted in the superior quadrants. The remaining 6 implants were placed in the inferior quadrants, including 3 in the fascia lata group (7.0%) and 3 in the corneal stroma group (7.3%).

**Table 1:** Patient characteristics in in the corneal stromal and fascia lata patches group. Intraoperatively, the part of the tube that lies on the sclera was covered with allogenous fascia lata or cornea. POAG: primary open angle glaucoma, PSXG: pseudoexfoliative glaucoma, GDI: glaucoma drainage device; AGI: Ahmed^®^ glaucoma implant, PGI: PAUL^®^ glaucoma implant.

	Corneal Stromal Patches	Fascia lata Patches	*p*-value
n	41	43	
Age [years]	57.3 ± 16.7	64.0 ± 17.3	0.589
Female	21 (51.2%)	18 (41.9%)	0.383
*Glaucoma Subtype*			
POAG	30 (73.2%)	27 (62.8%)	0.356
PSXG	3 (7.3%)	7 (16.3%)	0.314
Secondary Glaucoma	4 (9.8%)	8 (18.6%)	0.352
Congenital Glaucoma	4 (9.8%)	2 (4.7%)	0.427
*GDD*			
AGI	6 (14.6%)	26 (60.5%)	
PGI	35 (85.4%)	17 (39.5%)	

**Table 2:** Number and percentages of prior ocular surgeries in the corneal stromal and fascia lata patches group. SLT: selective laser trabeculoplasty, Kahook^®^: Kahook dual blade (New World Medical, Rancho Cucamonga, CA, USA), XEN^®^: XEN gel stent (Allergan inc., CA, USA), Preserflo^®^: Preserflo microshunt (MicroShunt, Santen, Munich, Germany, MS), iStent^®^: iStent trabecular micro-bypass stent (Glaukos corporation, Laguna Hills, CA, USA).

Ocular Surgery History	Corneal Stromal Patches	Fascia lata Patches
Phacoemulsification	32 (78.0%)	34 (79.1%)
Trabeculectomy	18 (43.9%)	22 (51.2%)
Trabeculectomy Revision	6 (14.6%)	6 (13.9%)
Cyclophotocoagulation	10 (24.4%)	7 (16.3%)
Vitrectomy	11 (26.8%)	15 (34.9%)
SLT	4 (9.8%)	8 (18.6%)
Canaloplasty	1 (2.8%)	1 (2.4%)
Trabeculotomy	2 (4.9%)	3 (7.0%)
Kahook^®^ Goniotomy	5 (12.2%)	4 (9.3%)
XEN^®^ Stent	4 (9.8%)	3 (7.0%)
Preserflo^®^ Stent	3 (7.3%)	1 (2.3%)
iStent^®^	2 (4.9%)	3 (7.0%)
Keratoplasty	4 (9.8%)	4 (9.3%)

**Fig. 1:**
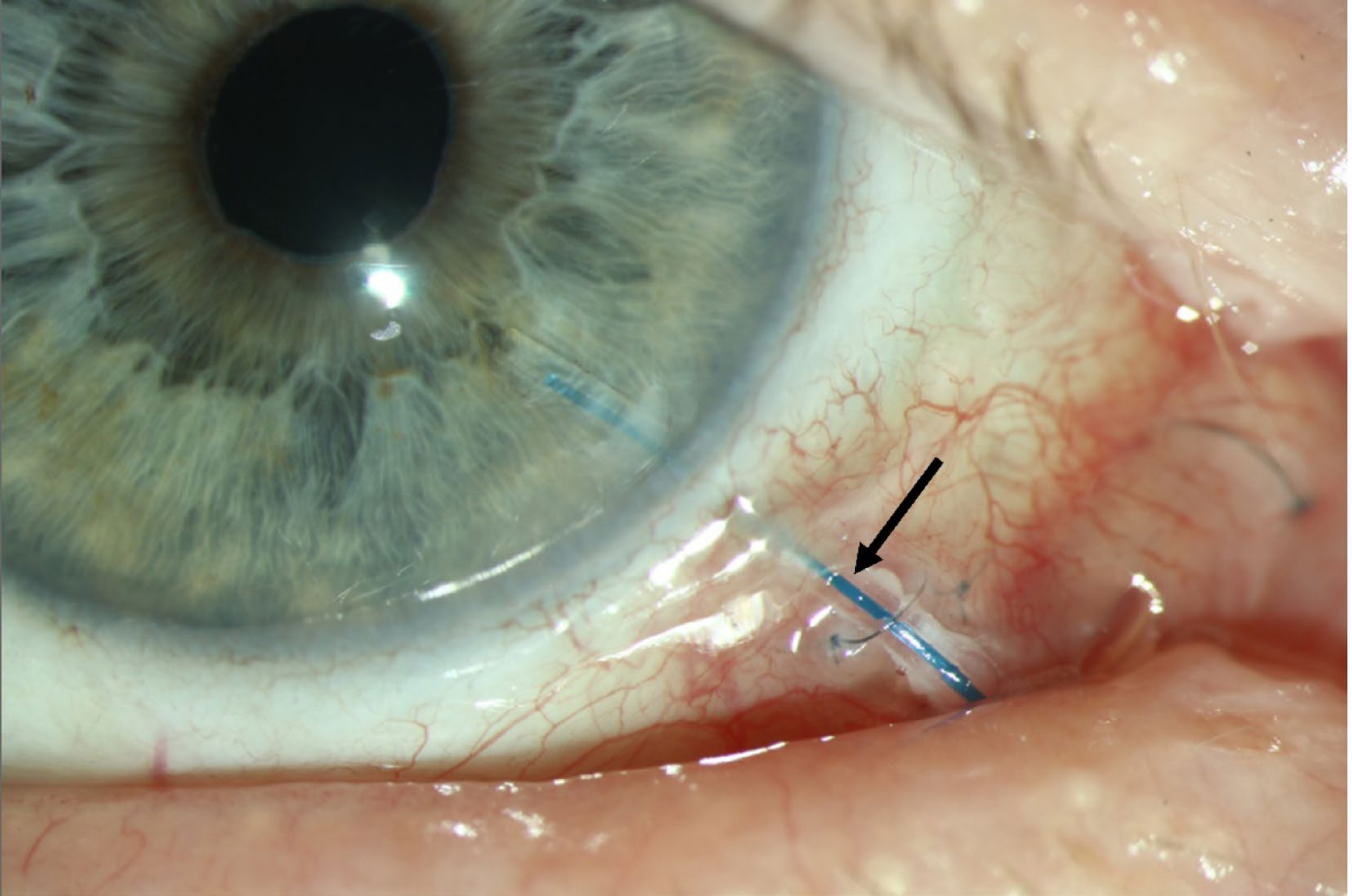
Tube erosion (arrow) 5.1 months following Paul glaucoma implant surgery with fascia lata patch in a 75-year-old male patient.

**Fig. 2:**
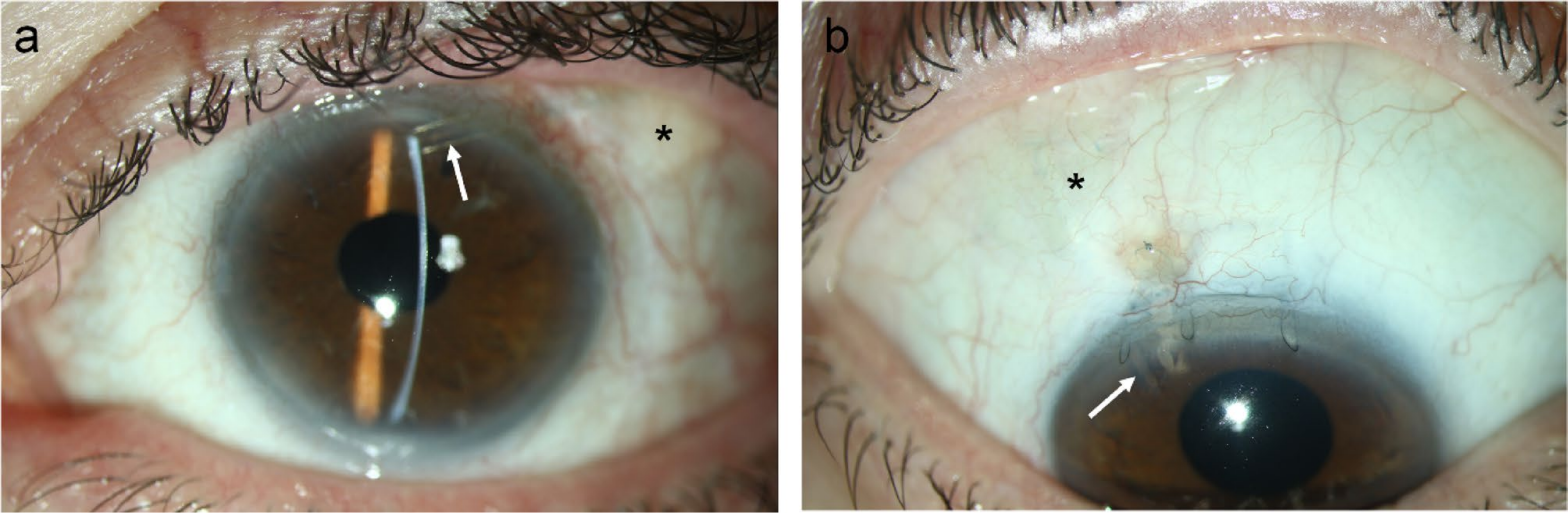
Appearance of a patient following Ahmed glaucoma implant surgery with a corneal stroma patch (**a**) and Paul glaucoma implant surgery with a fascia lata patch (**b**). The arrows point to the tubes, the asterisks (*) mark the position of the patch.

The mean follow-up in patients with fascia lata grafts was 14.1 ± 4.7 months (median 18.0 months) and 13.8 ± 5.0 months (median 12.0 months) in the corneal patch group (*p* = 0.270). After a mean follow-up of 9.2 ± 6.1 months (10.3 ± 6.5 months in the fascia lata group and 5.1 months in the corneal stroma group), tube erosions occurred in 4 patients (9.3%) with fascia lata patches and 1 patient (2.4%) with a corneal patch (*p* = 0.184, Fig. 1). In the superior quadrants, tube erosions were significantly more frequent in patients with fascia lata (*n* = 4) than corneal stromal patches (*n* = 0, *p* = 0.045). There were no differences in the number of tube erosions between the AGI (*n* = 2, 6.3%) and PGI (*n* = 3, 5.8%) groups (*p* = 0.928). No case of tube erosion was associated with severe complications, such as hypotony or endophthalmitis. Figure 2 shows the appearance of patients with a corneal stromal patch and a fascia lata patch in the superior quadrants.

**Fig. 3:**
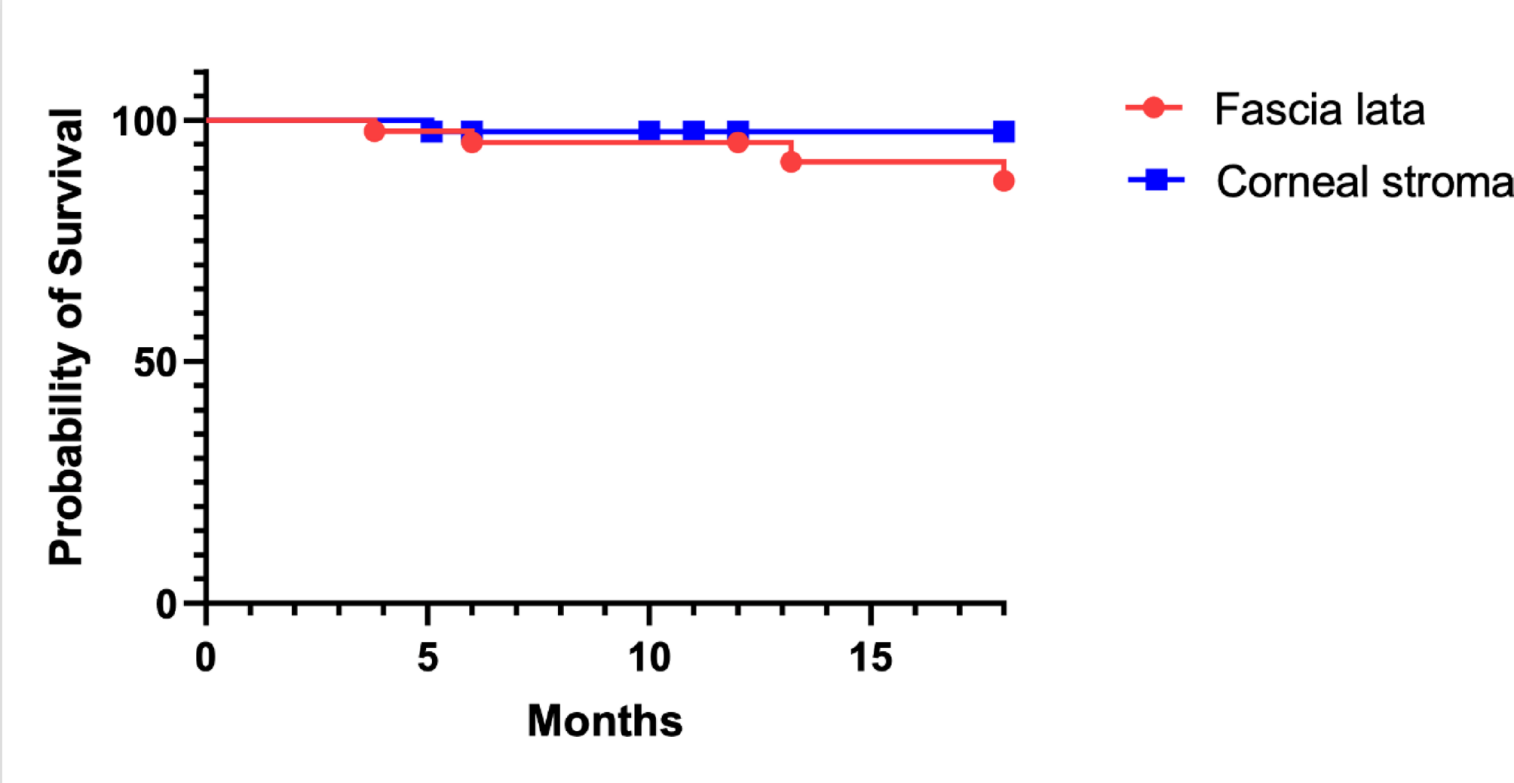
Kaplan–Meier analysis of tube patch survival over 18 months in fascia lata and corneal stroma patch groups.

A Kaplan–Meier survival analysis was conducted to assess the time to tube erosion in both patch graft groups. At 18 months, the estimated survival proportion was 87.4% in the fascia lata group and 97.6% in the corneal stroma group (overall survival rate: 91.8%). The difference between the groups was not statistically significant (log-rank *p* = 0.233). The survival curves are presented in Fig. 3.

In all 5 patients, the tube was re-covered with another patch and the conjunctiva was re-adapted. In 3 of these 5 (60.0%) patients with tube erosion, the tube was eroded again in the further postoperative course (Table 3). In 2 patients, the conjunctiva was rigid and could not be mobilized again. We therefore decided to explant the GDD in these patients.

**Table 3:** Characteristics of patients undergoing tube erosion after glaucoma drainage device (GDD) implantation. AGI: Ahmed^®^ glaucoma implant, PGI: PAUL^®^ glaucoma implant. M: male, F: female, POAG: primary open angle glaucoma, PSXG: pseudoexfoliative glaucoma, CPC: Cyclophotocoagulation.

Patient Number	Patch	Location	GDD	Age [Years]	Sex	Glaucoma Subtype	Previous Surgeries		Time to Tube Erosion [Months]		Re-Erosion?
1 (Fig. 1)	Corneal Stroma	Nasal Inferior	PGI	75	M	POAG	Phacoemulsification Trabeculectomy Trabeculectomy Revision (2)		5.1		14.5 months postoperatively, re-coverage with a new patch
2	Fascia Lata	Temporal Superior	PGI	80	F	POAG	Phacoemulsification Vitrectomy		18.0		–
3	Fascia Lata	Temporal Superior	AGI	55	F	POAG	Phacoemulsification Trabeculetomy		3.8		–
4	Fascia Lata	Temporal Superior	AGI	58	F	PSXG	Phacoemulsification Canaloplasty Trabeculectomy CPC		13.2		15.2 months postoperatively, leading to AGI explantation
5	Fascia Lata	Temporal Superior	PGI	72	M	Secondary glaucoma	Phacoemulsification CPC		6.0		8.3 months postoperatively, leading to PGI explantation

Preoperatively, intraocular pressure (IOP) was comparable between the AGI (31.9 ± 8.8 mmHg) and PGI groups (28.3 ± 10.1 mmHg; *p* = 0.096). The number of IOP-lowering medications was also similar at baseline (AGI: 3.5 ± 1.3; PGI: 3.2 ± 1.2; *p* = 0.295). At the 18-month follow-up, IOP was significantly reduced in both groups, reaching 15.8 ± 4.5 mmHg in the AGI group and 13.0 ± 3.8 mmHg in the PGI group (each *p* < 0.001 vs. baseline). Likewise, the number of IOP-lowering medications decreased significantly to 0.9 ± 1.1 in the AGI group and 0.5 ± 0.9 in the PGI group (each *p* < 0.001 vs. baseline).

**Fig. 4:**
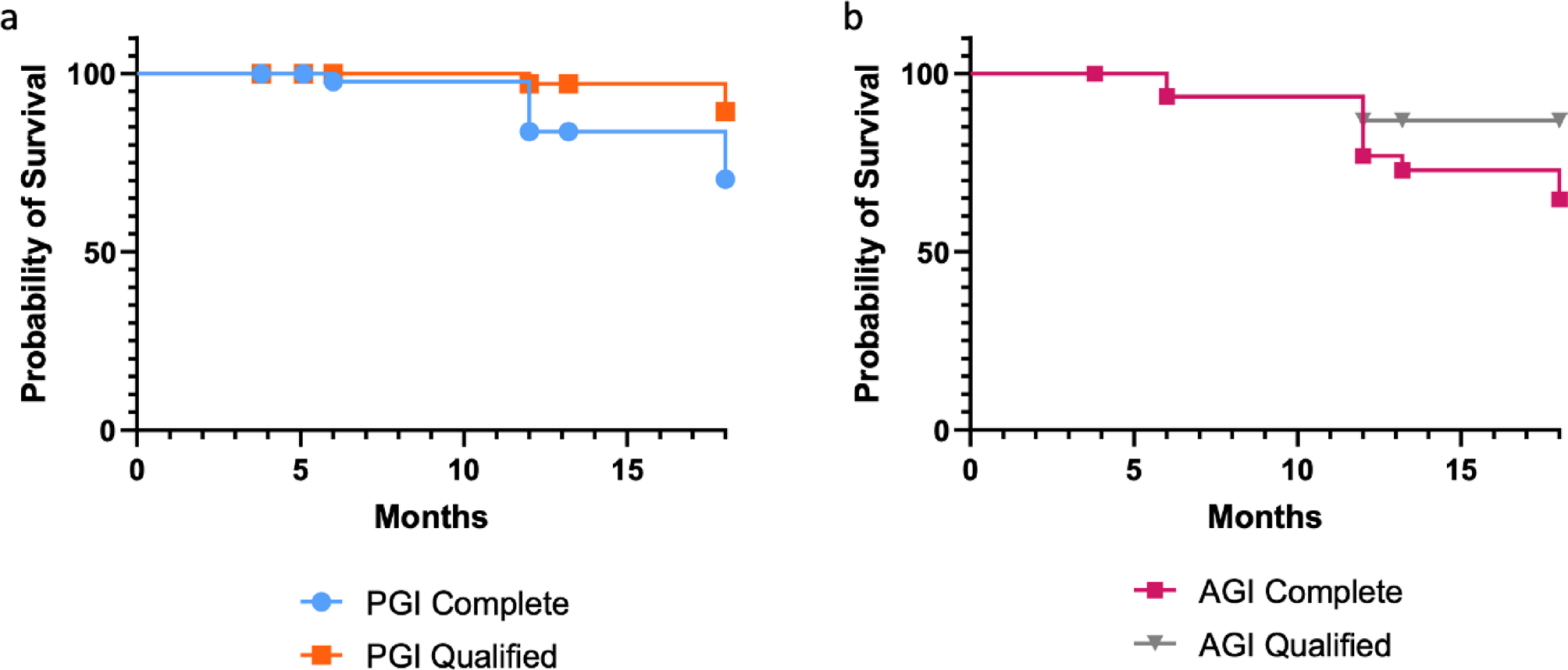
Kaplan–Meier survival curves for complete and qualified success (IOP ≤ 21 mmHg) over 18 months for the Paul glaucoma drainage implant (PGI, a) and Ahmed glaucoma drainage implant (AGI, b) groups.

At the 18-month follow-up, the complete success rate, defined as IOP ≤ 21 mmHg without medication, was 70.4% in the PGI group and 64.7% in the AGI group (*p* = 0.508, Fig. 4). Qualified success, defined as IOP ≤ 21 mmHg with or without medication, was achieved in 89.4% of PGI eyes and 86.9% of AGI eyes (*p* = 0.241).

## Discussion

According to the main findings of the present study, tube erosions are a rare complication after GDD. There were no differences in erosion rates between AGI and PGI. In the superior quadrants, fascia lata patches were significantly more often associated with tube erosions compared to corneal patches. The mean postoperative time to tube erosion was 9.2 ± 6.1 months.

This study focused on cases of postoperative tube erosion following GDD. Early patch or tube erosion within the first days to weeks after GDD implantation may be due to dehiscence of the conjunctival sutures rather than erosion of the conjunctiva^23^. Tube erosion through the conjunctiva may occur weeks to months after GDD implantation^28^. Loss of conjunctival vessels in the area over the tube seen during follow-up examinations may be an indication of impending erosion^28^. In our study, tube erosions occurred at a mean follow-up of 9.2 ± 6.1 months. Previous studies using bovine pericardial^17,29,30^allogenous corneal^17^or allogenous scleral patches^17^ reported a mean postoperative time to tube erosion ranging between 4 and 77 months^31^. To the best of our knowledge, data on tube erosions after fascia lata patches have not yet been published. Hypothetically, differences in patient characteristics and risk factors, as well as different patch materials, might account for the large differences in postoperative times to tube erosion.

A previous meta-analysis including 3,255 patients found a mean incidence of tube erosion of 2.0 ± 2.6% at a mean follow-up of 26.1 ± 3.3 months^12^. There were no differences in the incidence of tube erosions between the different GDD, including AGI, Baerveldt, and Molteno GDD^12^. No patients undergoing PGI were included in this meta-analysis^12^. The PGI has a smaller external and internal tube diameter (467 μm and 127 μm, respectively) than the AGI (635 μm and 305 μm) which hypothetically reduces the risk of tube erosion^25^. Current evidence suggests a tube erosion rate ranging between 0 and 4.1% following PGI using bovine pericardial patches to cover the tube^26,32^. However, in our study, there were no significant differences in erosion rates between AGI (6.3%) and PGI (5.8%) covered by either fascia lata or corneal stromal patches.

Also, the meta-analysis by Stewart et al. and further previous studies found that different patch materials did not affect the incidence of tube erosions^12,15,17^. However, only allogenous sclera, dura mater, and bovine pericardial patches were included in these studies. Wigton et al. found a significantly higher tube erosion rate in tubes covered with bovine pericardial compared to corneal patches^33^. To date, no fascia lata patches were included in any comparative studies^17,33^.

A worldwide shortage of donor corneas and dependence on cornea banking is a major limitation of corneal patches^34^. At our institution, donor corneas are provided by the in-house cornea bank. Also, we only used anterior lamellae from donor corneas which already served as graft tissue for DMEK surgery to use existing resources reasonably. Commercially available allografts or xenografts have become widely accepted for various conditions in ophthalmic surgery^35^including tube coverage in GDD surgery^30^. Their advantages mainly include uniformity in size and quality without dependence on cornea banking^30^. Also, the process designed to preserve the tissue for implantation involves dehydration and low-dose sterilization leading to an inactivation of infectious pathogens, such as Creutzfeld-Jakob virus and human immunodeficiency virus^36^.

In our study, we used full-thickness corneal stromal patch grafts without endothelium, prepared by trephining a central corneal button and removing the Descemet membrane. This technique differs from the split-thickness corneal patch grafts employed in other studies, where the anterior stroma is often dissected to create a thinner, lamellar graft. Spierer et al. reported a 6.7% rate of graft melting and a 2.2% rate of tube exposure using partial-thickness corneal grafts in a cohort of 45 eyes with a minimum follow-up of one year^37^.

In our cohort, the use of full-thickness corneal grafts may have hypothetically provided enhanced mechanical stability and resistance to melting or retraction, potentially contributing to the low erosion rate observed in the corneal patch group (1 out of 41 eyes, 2.4%). However, thicker grafts may increase graft visibility and thickness, possibly impacting cosmetic outcomes and patient comfort. Thinner split-thickness grafts might hypothetically offer improved cosmetic results and better patient tolerance but could be more prone to erosion due to reduced mechanical strength. Importantly, in our study, none of the patients reported any cosmetic concerns or discomfort related to graft visibility or appearance. To date, no studies have directly compared the clinical outcomes of full-thickness versus split-thickness corneal patch grafts for tube coverage, and future research is warranted to address this gap. Therefore, the choice of graft thickness and preparation technique should be individualized, balancing the risks of erosion with cosmetic considerations and patient-specific factors.

Fascia lata allografts have been explored as an alternative patch graft material in GDD surgery. The rationale for using fascia lata grafts for tube coverage lies in their structural integrity, biocompatibility, and availability as allografts^35^. Their collagen-rich composition and flexibility may offer sufficient initial mechanical support to protect the tube from erosion, particularly when other graft materials are unavailable or unsuitable. However, their long-term structural integrity remains a concern. A previous study employed allogenous fascia lata grafts to lengthen eye muscles in patients with Graves’ orbitopathy. Histological examination revealed that the fascia lata grafts were indistinguishable from the surrounding tissue after less than 1 year postoperatively^38^. Also, fascia lata allografts used in urogynaecologic surgeries postoperatively showed areas of disorganized remodeling and graft degeneration in histological examinations^39^. In our study, fascia lata grafts appeared to be more frequently associated with tube erosions compared to corneal grafts. Therefore, and based on previous histological findings, we hypothesize that allogenous fascia lata grafts show a higher tendency to dissolve after implantation into the host body compared to corneal patches, which might be disadvantageous for the purpose of tube coverage. This may limit their capacity to provide sustained mechanical coverage over GDD tubes, particularly under conditions of constant ocular surface exposure and friction.

In contrast, corneal tissue—adapted to withstand environmental stressors such as desiccation or shear forces—may offer superior long-term resistance to erosion when used as a patch graft. Future prospective comparative studies with large patient cohorts and long-term follow-up are warranted to systematically evaluate the biomechanical properties, degradation profiles, and clinical outcomes associated with different patch graft materials in GDD surgery.

In a study by Tanji et al., 22 eyes received GDDs with fascia lata patch grafts. The authors positioned the grafts such that the parallel fibres were oriented perpendicular to the tube axis, aiming to prevent splitting of the material. Over a mean follow-up period of 19 months, no cases of tube exposure were observed^22^. In our cohort, we applied the same graft orientation technique but still observed a tube erosion rate of 9.3% in the fascia lata group. Potential explanations for this difference include variations in patient populations and baseline characteristics, surgical indications, and postoperative management, as well as subtle differences in graft handling and fixation. Notably, a considerable proportion of our patients had undergone previous ocular surgeries, potentially compromising conjunctival integrity and wound healing.

To date, the exact risk factors for tube erosion are still largely unknown. However, immune-mediated inflammation, insufficient conjunctival perfusion leading to conjunctival ischemia, pronounced conjunctival tissue tension, eyelid trauma, Hispanic ethnicity, neovascular glaucoma, and young age have been identified to contribute to conjunctival erosion following GDI surgery^29,40–43^. However, in our study, no such correlations were calculated given the limited sample sizes. Moreover, previous trabeculectomy surgery was identified as a risk factor for tube erosion following GDD^29,43^. In our study, only one patient with corneal patch tube coverage was diagnosed with a tube erosion. This patient previously underwent multiple surgeries, including trabeculectomy and 2 trabeculectomy revision surgeries with subsequent excessive scarring of the superior conjunctiva. An increased number of fibroblasts, macrophages, and lymphocytes in the conjunctival substantia propria of patients with previous conjunctival incisional procedures compared to patients without prior ocular surgery has been reported^44^. Therefore, the conjunctival microenvironment might be primed for an excessive immune-mediated response at the time of repeat surgery, which is a known risk factor for tube erosion^29,44^.

Also, the PGI was placed in the infero-nasal quadrant in our patient with tube erosion following corneal patch tube coverage. Levinson et al. found a 3-times higher tube erosion rate for GDD implanted inferiorly compared to the supero-temporal quadrant^17^. The highest rate of tube erosions was reported for the infero-nasal quadrant (17.2%). Hypothetically, this is attributable to higher exposure of the implant due to a shorter inferior fornix compared to a superior location^17,45^.

Mitomycin C was routinely applied intraoperatively in all cases, regardless of the type of GDD (PGI or AGI) or the patch graft material used (fascia lata or corneal stroma). Mitomycin C is known for its antifibrotic effect, which can reduce postoperative scarring and improve surgical outcomes in glaucoma procedures. However, it may also impair conjunctival healing and has been implicated in increasing the risk of complications such as graft thinning or tube exposure. Since mitomycin C was used uniformly across all groups in our study, any potential impact would have most likely affected all cases equally. Therefore, it appears unlikely that mitomycin C contributed to the observed differences in tube erosion rates between the two patch graft types.

This study is limited by its number of patients lost to follow-up. In our patient cohort, the mean follow-up time to tube erosion was 9 months, and there was one case of tube erosion at the final follow-up appointment of 18 months postoperatively. More cases of tube erosion could occur after the final follow-up appointment. This must be taken into account when interpreting the results of this study. However, as a tertiary centre, follow-up periods are usually limited in patients with uneventful postoperative courses. Therefore, it can be assumed that patients with any severe complications requiring surgery, such as tube erosion, would have been referred to our institution by their local ophthalmologists.

Moreover, patients undergoing AGI and PGI were included in this study. The different tube diameters of AGI and PGI hypothetically influence the incidence of tube erosions and may confound the results of fascia lata vs. corneal stromal patches.

A potential limitation of our study is the non-randomized, retrospective design, which may introduce selection bias. The choice of GDD (AGI vs. PGI) and patch graft material (fascia lata vs. corneal stroma) was not standardized but determined by the operating surgeon based on graft availability, handling characteristics, and individual patient factors. Notably, fascia lata was predominantly used earlier in the study period, whereas corneal stroma became more frequently applied in later cases due to improved access and favourable surgical properties. While baseline characteristics between the groups were largely comparable, the non-randomized allocation could have influenced the observed outcomes. Future prospective studies with randomized graft allocation are needed to confirm our findings and minimize selection-related confounding factors.

Also, the small number of patients with tube erosion possibly limits the statistical power of the analysis. However, to the best of our knowledge, this is the first study comparing fascia lata to corneal stromal patches to cover the tubes.

## Conclusion

According to the findings of the present study, tube erosions are a rare complication after GDD implantations. However, tubes covered with fascia lata were associated with higher erosion rates compared to corneal stromal patches.

## Data Availability

The datasets generated during and/or analysed during the current study are available throughout the manuscript.
